# Supplementary material for: An auxin-inducible, GAL4-compatible, gene expression system for Drosophila
Source: eLife. 2022 Apr 1;11:e67598. doi: 10.7554/eLife.67598 (PMC8975555; doi:10.7554/eLife.67598)
Supplement: Figure 1—figure supplement 1—source data 1. [file elife-67598-fig1-figsupp1-data1.zip › 67598Figure1FIgureSupplement1SourceData1]

```
LOCUS       Exported               13306 bp ds-DNA     circular SYN 19-
MAR-2019
DEFINITION  synthetic circular DNA
ACCESSION   .
VERSION     .
KEYWORDS    .
SOURCE      synthetic DNA construct
  ORGANISM  synthetic DNA construct
REFERENCE   1  (bases 1 to 13306)
  AUTHORS   Southall lab
  TITLE     Direct Submission
  JOURNAL   Exported Thursday, Jan 21, 2021 from SnapGene Viewer 5.0.7
            https://www.snapgene.com
FEATURES             Location/Qualifiers
     source          1..13306
                     /organism="synthetic DNA construct"
                     /mol_type="other DNA"
     misc_feature    83..2677
                     /label=Tubulin Promoter
     CDS             2683..4464
                     /codon_start=1
                     /label=AtTIR1

/translation="MQKRIALSFPEEVLEHVFSFIQLDKDRNSVSLVCKSWYEIERWCR

RKVFIGNCYAVSPATVIRRFPKVRSVELKGKPHFADFNLVPDGWGGYVYPWIEAMSSSY

TWLEEIRLKRMVVTDDCLELIAKSFKNFKVLVLSSCEGFSTDGLAAIAATCRNLKELDL

RESDVDEVSGHWLSHFPDTYTSLVSLNISCLASEVSFSALERLVTRCPNLKSLKLNRAV

PLEKLATLLQRAPQLEELGTGGYTAEVRPDVYSGLSVALSGCKELRCLSGFWDAVPAYL

PAVYSVCSRLTTLNLSYATVQSYDLVKLLCQCPKLQRLWVLDYIEDAGLEVLASTCKDL

RELRVFPSEPFVMEPNVALTEQGLVSVSMGCPKLESVLYFCRQMTNAALITIARNRPNM

TRFRLCIIEPKAPDYLTLEPLDIGFGAIVEHCKDLRRLSLSGLLTDKVFEYIGTYAKKM

EMLSVAFAGDSDLGLHHVLSGCDSLRKLEIRDCPFGDKALLANASKLETMRSLWMSSCS

VSFGACKLLGQKMPKLNVEVIDERGAPDSRPESCPVERVFIYRTVAGPRFDMPGFVWNM
                     DQDSTMRFSRQIITTNGL"
     misc_feature    4465..4467
                     /label=GSG Link
     misc_feature    4468..4521
                     /label=T2A
     misc_feature    4522..4653
                     /label=mini_AID
     primer_bind     4627..4687
                     /label=dGAL80_FW
     CDS             4657..5958
```

eLife Sciences Publications, Ltd is a limited liability non-profit nonstock corporation incorporated in the State of Delaware, USA with company number 5030732, and is registered in the UK with company number FC030576 and branch number BR015634 at the address Westbrook Centre, Milton Road, Cambridge, CB4 1YG, UK.

```
                     /codon_start=1
                     /label=dGAL80

/translation="DYNKRSSVSTVPNAAPIRVGFVGLNAAKGWAIKTHYPAILQLSSQ

FQITALYSPKIETSIATIQRLKLSNATAFPTLESFASSSTIDMIVIAIQVASHYEVVMP

LLEFSKNNPNLKYLFVEWALACSLDQAESIYKAAAERGVQTIISLQGRKSPYILRAKEL

ISQGYIGDINSIEIAGNGGWYGYERPVKSPKYIYEIGNGVDLVTTTFGHTIDILQYMTS

SYFSRINAMVFNNIPEQELIDERGNRLGQRVPKTVPDHLLFQGTLLNGNVPVSCSFKGG

KPTKKFTKNLVIDIHGTKGDLKLEGDAGFAEISNLVLYYSGTRANDFPLANGQQAPLDP

GYDAGKEIMEVYHLRNYNAIVGNIHRLYQSISDFHFNTKKIPELPSQFVMQGFDFEGFP
                     TLMDALILHRLIESVYKSNMMGSTLNVSNISHYSL"
     primer_bind     complement(5928..5958)
                     /label=dGAL80_RV
     misc_feature    5971..6105
                     /label=dAID
     primer_bind     complement(6027..6133)
                     /label=OligoB
     primer_bind     complement(6142..6164)
                     /label=UAS_Rv
     intron          6192..6257
                     /label=small t intron
                     /note="simian virus 40 (SV40) small t antigen
intron"
     CDS             6387..6407
                     /codon_start=1
                     /product="nuclear localization signal of SV40 large
T
                     antigen"
                     /label=SV40 NLS
                     /translation="PKKKRKV"
     polyA_signal    6679..6813
                     /label=SV40 poly(A) signal
                     /note="SV40 polyadenylation signal"
     CDS
complement(join(7829..8443,8514..8645,8849..9164,9226..9880,
                     9955..10228,10630..10701))
                     /codon_start=1
                     /gene="white"
                     /product="Drosophila white gene eye color pigment"
                     /label=mini-white
                     /note="This is a modified version of the white gene
lacking
                     part of the first intron."

/translation="MGQEDQELLIRGGSKHPSAEHLNNGDSGAASQSCINQGFGQAKNY

GTLRPPSPPEDSGSGSGQLAENLTYAWHNMDIFGAVNQPGSGWRQLVNRTRGLFCNERH
```

eLife Sciences Publications, Ltd is a limited liability non-profit nonstock corporation incorporated in the State of Delaware, USA with company number 5030732, and is registered in the UK with company number FC030576 and branch number BR015634 at the address Westbrook Centre, Milton Road, Cambridge, CB4 1YG, UK.

```
IPAPRKHLLKNVCGVAYPGELLAVMGSSGAGKTTLLNALAFRSPQGIQVSPSGMRLLNG

QPVDAKEMQARCAYVQQDDLFIGSLTAREHLIFQAMVRMPRHLTYRQRVARVDQVIQEL

SLSKCQHTIIGVPGRVKGLSGGERKRLAFASEALTDPPLLICDEPTSGLDSFTAHSVVQ

VLKKLSQKGKTVILTIHQPSSELFELFDKILLMAEGRVAFLGTPSEAVDFFSYVGAQCP

TNYNPADFYVQVLAVVPGREIESRDRIAKICDNFAISKVARDMEQLLATKNLEKPLEQP

ENGYTYKATWFMQFRAVLWRSWLSVLKEPLLVKVRLIQTTMVAILIGLIFLGQQLTQVG

VMNINGAIFLFLTNMTFQNVFATINVFTSELPVFMREARSRLYRCDTYFLGKTIAELPL

FLTVPLVFTAIAYPMIGLRAGVLHFFNCLALVTLVANVSTSFGYLISCASSSTSMALSV

GPPVIIPFLLFGGFFLNSGSVPVYLKWLSYLSWFRYANEGLLINQWADVEPGEISCTSS

NTTCPSSGKVILETLNFSAADLPLDYVGLAILIVSFRVLAYLALRLRARRKE"
     protein_bind    11396..11465
                     /label=attB
                     /bound_moiety="phage phi-C31 integrase"
                     /note="attB site for the phi-C31 integrase (Groth
et al.,
                     2000)"
     rep_origin      complement(11552..12140)
                     /direction=LEFT
                     /label=ori
                     /note="high-copy-number ColE1/pMB1/pBR322/pUC
origin of
                     replication"
     CDS             complement(12311..13171)
                     /codon_start=1
                     /gene="bla"
                     /product="beta-lactamase"
                     /label=AmpR
                     /note="confers resistance to ampicillin,
carbenicillin, and
                     related antibiotics"

/translation="MSIQHFRVALIPFFAAFCLPVFAHPETLVKVKDAEDQLGARVGYI

ELDLNSGKILESFRPEERFPMMSTFKVLLCGAVLSRIDAGQEQLGRRIHYSQNDLVEYS

PVTEKHLTDGMTVRELCSAAITMSDNTAANLLLTTIGGPKELTAFLHNMGDHVTRLDRW

EPELNEAIPNDERDTTMPVAMATTLRKLLTGELLTLASRQQLIDWMEADKVAGPLLRSA

LPAGWFIADKSGAGERGSRGIIAALGPDGKPSRIVVIYTTGSQATMDERNRQIAEIGAS
                     LIKHW"
     promoter        complement(13172..13276)
                     /gene="bla"
```

eLife Sciences Publications, Ltd is a limited liability non-profit nonstock corporation incorporated in the State of Delaware, USA with company number 5030732, and is registered in the UK with company number FC030576 and branch number BR015634 at the address Westbrook Centre, Milton Road, Cambridge, CB4 1YG, UK.

```
                     /label=AmpR promoter
ORIGIN
        1 taagaaacca ttattatcat gacattaacc tataaaaata ggcgtatcac
gaggcccttt
       61 cgtcttcaag gaattcgata tcaagcttgc acaggtcctg ttcgataacg
tcgtactcgg
      121 gaatcacctt taacttgtcc gctattgggt atttgtcctt aaacacattc
gtaatctctc
      181 ccacaatcat tgccctggcc tctgcaatgt aaaaagtatt tataaaatat
cgctggctct
      241 aaaggaaaca tattaatcaa agtattgagg ctttatcaat ctaacattta
caagtagtca
      301 gaccacaatt tttagactgc acttctgcgt aagtaaatga aatgaaatgg
tgacctactc
      361 ttgggtagcc gtactgttat atgctgacga gccttcacct gggcctgctt
tttctcctgt
      421 ttcgtctgat gcgtcggatg cggctggact ggctgattgt tgggattggg
ttttggatgc
      481 tgctccgaaa ggtgggccac agcttttgcg atggtggtgc ccactatgcg
gttgctgcag
      541 gaactacagc tgaatgttcg ctttattgtc tgcagctcct ggggctggac
gaaggccacg
      601 ccctcacccg gtgcggccat ctcctgcggc aaaacgtatg tgggattcct
gtccaagtag
      661 ctctcgatgc ccaccataat gcgctccagc aggttggcca ggtgcgggtc
tagctgcggc
      721 ggattcgtcg tctggttcac tgagtttagg gtctgcagaa agaagtgtgc
ctccatggta
      781 agcggattca tctgcacgat cagcgggctt tagtctaaaa attcgaatga
tacaaactcg
      841 tactcacgta aagcagtttg tttggcatca tctcggccat cgtcttattc
ttcttcgtgg
      901 cctttgttac cggagcggct atgttgggcg cggtggagcc cggaagctga
aaggttaatc
      961 gaatgcgtta acttttctgc aactcgaaag tttgccgcct ttgttcgact
gccaataact
     1021 gttgattcga aaattcgaat cgaagcggtt gaatttcgta gggtggccaa
ctacaccaaa
     1081 gttcgccggc ggtgtatgca tgtgagtgtg agtgtgacag tgggtgtacg
tgtgctttaa
     1141 gcgacacaat cgcaccgcga tgtacatatt tttgtagtaa tgtgtgtgca
tttttcgtcc
     1201 ggatcacggc tccataaaat aaatcatatc acagggctca gggatttgtg
agtagaagtt
     1261 cccgccactt aaacgtcact tgtgaaatcg ccatcgacgg tcaactttta
ctcacactgg
     1321 tctttcgcgg acggaccgtc tcaaagtact gcctttctgc gttggaaaac
atcgcctttt
     1381 tcgtccaaaa ggagtcccca ggttcgatcc gcatggcgtt gtgcgtgcgt
gcctttcttt
     1441 tcaaatgatt acggctatta acttgggggc gttaagttgg aaacacgtaa
attgcagact
     1501 gcgattagag tgaccatgag taggagttca aaatctcctg acatcatttt
```

eLife Sciences Publications, Ltd is a limited liability non-profit nonstock corporation incorporated in the State of Delaware, USA with company number 5030732, and is registered in the UK with company number FC030576 and branch number BR015634 at the address Westbrook Centre, Milton Road, Cambridge, CB4 1YG, UK.

cttaaaacct
1561 gctttgtttt ttacatttct atttaatata actcctattt gaataaaaaa acaaaacaag
1621 tttagatgtt aagatattaa ctacatcctt tgctccaaag ggagagggga agttatggag
1681 ttaattaatt tgctgttgga aatcaatatg gagtcagaaa tataatgatt tactaaacct
1741 tattgaatcg gtaacgatgc gaatttatat taaaatagct tttatgaaac attcaacaaa
1801 aatattatta atgttggccc actttagcaa ccggttaggt ctaccggttg ggcaagcaaa
1861 gattcacgcc ctggttcgag tcccaactag tcctgcaaaa taccgcagca agttttagag
1921 agaccaagtg ccattacctc tcccacttca gttatcggtt atgcggcgtt taagtcgaca
1981 gcttgccgtc tctagctccg gtgcctatat aaagcagccc gctttccaca tttcatattc
2041 gttttacgtt tgtcaagcct catagccggc agttcgaacg tatacgctct ctgagtcaga
2101 cctcgaaatc gtagctctac acaattctgt gaattttcct tgtcgcgtgt gaaacacttc
2161 caataaaaac tcctgcaggt gagtacttta aaaaaaaatc tagtgaaata atgctgaaaa
2221 gaaatttgtg tgggcaaaat tcaatgggca aaaacgcgat gcggcttttt ctcaaaatgg
2281 cggccggcct gcgtttttc ctcaaaagtg atgacgtcat gcctgttttt tttttttgt
2341 tcgcaatgag gaatggctct taaaatctac tagataaaaa aaatattcat tatttctatg
2401 ctgctggaac gcttcattaa tcttaaaaat tctaaattcg gttaccatga tacttcgacg
2461 cataactgta gattttggat agaattaaag agaaaatggc gagagagtaa aattccggcg
2521 tcggcaaagt agagcaaaaa aatcagtata ccatttagct acctctctca ctcgcacgca
2581 gtgccggctc aagttgggcg cggctctgca attatcgatt ttcttggggt gtgtaactaa
2641 tcatccgttt tcccttcctc ctcatccaca gcgtgaaggt acatgcaaaa gcgcatagca
2701 ttgagcttcc ctgaggaagt actggagcac gtattttcct tcatccagct cgataaggac
2761 aggaatagtg tgtccctggt atgtaaaagc tggtacgaga tcgagcgatg gtgcaggcgc
2821 aaggtcttca ttggaaactg ctacgctgtg agtcccgcaa ccgtaatacg tcgcttcccg
2881 aaagtgcgaa gtgtggagct caagggtaaa ccacatttcg ctgattttaa tttggttccg
2941 gacggatggg gaggctatgt ttatccctgg atcgaggcga tgagcagcag ttacacctgg
3001 ttggaggaga tacgtctgaa gcgtatggtc gtcactgacg attgcttgga actcatagct
3061 aaaagtttca agaacttcaa ggtcctggtc ctctccagct gcgagggttt ctcgacggat

eLife Sciences Publications, Ltd is a limited liability non-profit nonstock corporation incorporated in the State of Delaware, USA with company number 5030732, and is registered in the UK with company number FC030576 and branch number BR015634 at the address Westbrook Centre, Milton Road, Cambridge, CB4 1YG, UK.

3121 ggtctggccg caatcgccgc aacgtgccgg aacctcaagg aattggatct
ccgtgagagc
3181 gacgttgatg aggttagtgg tcactggctc agccactttc cagatacata
tacgagtctg
3241 gtctcgctga atatcagttg cctggcctcg gaagtatcct tcagcgctct
ggaacgtttg
3301 gtcactcgat gcccaaatct gaaatcgctc aaattgaaca gggcagtccc
cttggagaaa
3361 ttggcgaccc tgctccagcg cgctccgcag ttggaggaat tgggtacggg
cggttataca
3421 gcggaggttc gtcccgatgt ttactccggt ctgtcggttg cgctctcggg
ttgcaaagag
3481 ctcaggtgct tgtccggatt ttgggatgct gtcccggcgt acctgcccgc
cgtatactcg
3541 gtttgtagcc ggctgactac actgaatctg tcctatgcta cagtacaatc
ctacgacttg
3601 gttaaactgc tctgtcaatg tcccaaactc caacgcctct gggttctgga
ctatattgaa
3661 gacgctggac tcgaagtcct ggcctcgacg tgtaaagact tgcgcgagct
gcgcgtgttc
3721 ccctccgaac ctttcgtcat ggagcctaat gtagcactca ccgaacaggg
cttggtaagc
3781 gtttcgatgg gctgtcctaa gttggaatcg gtactctact tctgtcgtca
aatgacgaat
3841 gctgcgttga taacgatcgc acggaatagg cccaatatga cccggttcag
gttgtgtatt
3901 atcgaaccaa aggcccccga ttatttgacg ttggagcctt tggacatcgg
ctttggtgcg
3961 atagtcgaac attgcaaaga cctgcgacgc ttgtccctca gtggcctcct
gaccgacaag
4021 gttttcgagt acattggtac ttatgctaaa aagatggaga tgttgagtgt
ggcgtttgcc
4081 ggtgatagcg acctcggctt gcaccacgta ctgagcggtt gtgattcgct
gcgcaaactc
4141 gaaataaggg actgtccctt tggagataag gcgttgttgg cgaacgcgag
caaattggag
4201 accatgcgat ccttgtggat gtcgtcctgt agtgtaagct tcggcgcatg
caagttgctc
4261 ggccagaaaa tgccgaaact caacgtcgaa gttattgacg agcgaggcgc
tccagatagt
4321 aggcccgagt cctgtccagt cgagcgagtt ttcatatacc ggaccgtagc
aggaccccgc
4381 ttcgacatgc ctggcttcgt gtggaacatg gatcaagaca gtactatgcg
cttttcccga
4441 cagattataa caaccaatgg actcggcgag ggccgcggca gcctgctgac
ctgcggcgat
4501 gtggaggaga accccggccc ccctaaagat ccagccaaac ctccggccaa
ggcacaagtt
4561 gtgggatggc caccggtgag atcataccgg aagaacgtga tggtttcctg
ccaaaaatca
4621 agcggtggcc cggaggcggc ggcgttcgtg aagatggatt acaacaaaag
gagtagtgtg
4681 agtacggtgc cgaatgctgc tcccattcgc gtgggcttcg tgggattgaa

eLife Sciences Publications, Ltd is a limited liability non-profit nonstock corporation incorporated in the State of Delaware, USA with company number 5030732, and is registered in the UK with company number FC030576 and branch number BR015634 at the address Westbrook Centre, Milton Road, Cambridge, CB4 1YG, UK.

cgcggctaag
4741 ggttgggcca ttaaaacgca ttatccagcc atactgcagc tgagctccca gttccaaata
4801 acagcgcttt attcccccaa gatcgagacg tccattgcga caattcagag gctgaagttg
4861 tccaacgcca cagctttccc aacactcgag agcttcgcct cgagctccac gatcgatatg
4921 attgtgatcg caatccaggt ggcttcccac tacgaggtgg taatgcccct gctcgagttt
4981 tcgaagaaca atcctaatct gaagtatttg tttgtggaat gggcactggc ctgctcgctc
5041 gatcaggcgg agtcgatcta taaggccgct gcagagcggg gcgtgcaaac aataatcagt
5101 ctgcagggac gaaagagccc gtacattttg cgggcaaaag agcttatctc ccagggctac
5161 atcggcgata taaatagcat cgagattgca ggtaacggag gttggtacgg ttacgagcgg
5221 ccggtgaaaa gcccgaaata catttacgag atcggaaacg gagttgatct ggtgaccacc
5281 acgttcggtc acacgataga tatattgcag tacatgacca gttcgtactt cagccgtatc
5341 aatgccatgg tgtttaacaa cattccagaa caggagctca ttgatgagag gggcaatcgc
5401 ctgggccagc gggtcccaaa gactgtccca gatcatctct tgttccaggg cacgctgctc
5461 aacggaaatg ttcctgtgtc ctgttccttt aagggaggca agcctacgaa aaagttcacc
5521 aagaatctgg tcatagatat ccatggtaca aagggtgatc tgaagctgga gggtgacgct
5581 ggatttgcgg aaatctcgaa tctcgttctg tattattcgg gcacccgcgc caatgatttt
5641 cccctggcca atggtcagca ggcacccttg gaccccggct acgacgcagg caaggagatt
5701 atggaggtgt atcacctccg aaactacaac gcgatcgttg gtaatataca ccggctctac
5761 cagagtatca gcgatttcca cttcaacacc aagaaaatcc ccgagcttcc atcgcagttc
5821 gtaatgcaag gcttcgattt cgaaggcttt cccaccctga tggatgcctt gatcttgcat
5881 cgcctgattg aaagtgtcta caagtcgaat atgatgggca gtaccctgaa tgtttccaac
5941 atctcccact actcgcttgg tcctgcgatg cccaaggacc cagccaagcc acccgccaag
6001 gcccaggtgg tgggctggcc gcccgtgcgc tcctaccgca agaacgtgat ggtgtcctgc
6061 cagaagtcct ccggcggccc ggaggccgcc gccttcgtga agtagccttc tagaggatct
6121 ttgtgaagga accttacttc tgtggtgtga cataattgga caaactacct acagagattt
6181 aaagctctaa ggtaaatata aaatttttaa gtgtataatg tgttaaacta ctgattctaa
6241 ttgtttgtgt attttagatt ccaacctatg gaactgatga atgggagcag tggtggaatg

eLife Sciences Publications, Ltd is a limited liability non-profit nonstock corporation incorporated in the State of Delaware, USA with company number 5030732, and is registered in the UK with company number FC030576 and branch number BR015634 at the address Westbrook Centre, Milton Road, Cambridge, CB4 1YG, UK.

6301 cctttaatga ggaaaacctg ttttgctcag aagaaatgcc atctagtgat gatgaggcta
6361 ctgctgactc tcaacattct actcctccaa aaaagaagag aaaggtagaa gaccccaagg
6421 actttccttc agaattgcta agttttttga gtcatgctgt gtttagtaat agaactcttg
6481 cttgctttgc tatttacacc acaaaggaaa aagctgcact gctatacaag aaaattatgg
6541 aaaaatattt gatgtatagt gccttgacta gagatcataa tcagccatac cacatttgta
6601 gaggttttac ttgctttaaa aaacctccca cacctccccc tgaacctgaa acataaaatg
6661 aatgcaattg ttgttgttaa cttgtttatt gcagcttata atggttacaa ataaagcaat
6721 agcatcacaa atttcacaaa taaagcattt ttttcactgc attctagttg tggtttgtcc
6781 aaactcatca atgtatctta tcatgtctgg atccccgcgc ggccgctggc cacgggtgcg
6841 catgatcgtg ctcctgtcgt tgaggacccg gctaggctgg cggggttgcc ttactggtta
6901 gcagaatgaa tcaccgatac gcgagcgaac gtgaagcgac tgctgctgca aaacgtctgc
6961 gacctgagca acaacatgaa tggtcttcgg tttccgtgtt tcgtaaagtc tggaaacgcg
7021 gaagtcagcg ccctgcacca ttatgttccg gaggcgcgcc ctagttccag tgaaatccaa
7081 gcatttccta aattaaatgt attcttatta ttatagttgt tatttttgat atatataaac
7141 aacactatta tgcccaccat ttttttgaga tgcatctaca caaggaacaa acactggatg
7201 tcactttcag ttcaaattgt aacgctaatc actccgaaca ggtcacaaaa aattacctta
7261 aaaagtcata atattaaatt agaataaata tagctgtgag ggaaatatat acaaatatat
7321 tggagcaaat aaattgtaca tacaaatatt tattactaat ttctattgag acgaaatgaa
7381 ccactcggaa ccatttgagc gaaccgaatc gcgcggaact aacgacagtc gctccaaggt
7441 cgtcgaacaa aaggtgaatg tgttgcggag agcgggtggg agacagcgaa agagcaacta
7501 cgaaacgtgg tgtggtggag gtgaattatg aagagggcgc gcgatttgaa aagtatgtat
7561 ataaaaaata tatcccggtg ttttatgtag cgataaacga gtttttgatg taaggtatgc
7621 aggtgtgtaa gtcttttggt tagaagacaa atccaaagtc tacttgtggg gatgttcgaa
7681 ggggaaatac ttgtattcta taggtcatat cttgttttta ttggcacaaa tataattaca
7741 ttagcttttt gaggggcaa taaacagtaa acacgatggt aataatggta aaaaaaaaaa
7801 acaagcagtt atttcggata tatgtcggct actccttgcg tcgggcccga agtcttagag
7861 ccagatatgc gagcacccgg aagctcacga tgagaatggc cagacccacg

eLife Sciences Publications, Ltd is a limited liability non-profit nonstock corporation incorporated in the State of Delaware, USA with company number 5030732, and is registered in the UK with company number FC030576 and branch number BR015634 at the address Westbrook Centre, Milton Road, Cambridge, CB4 1YG, UK.

tagtccagcg
7921 gcagatcggc ggcggagaag ttaagcgtct ccaggatgac cttgcccgaa
ctggggcacg
7981 tggtgttcga cgatgtgcag ctaatttcgc ccggctccac gtccgcccat
tggttaatca
8041 gcagaccctc gttggcgtaa cggaaccatg agaggtacga caaccatttg
aggtatactg
8101 gcaccgagcc cgagttcaag aagaagccgc caaagagcag gaatggtatg
ataaccggcg
8161 gacccacaga cagcgccatc gaggtcgagg agctggcgca ggatattaga
tatccgaagg
8221 acgttgacac attggccacc agagtgacca gcgccaggca gttgaagaag
tgcagcactc
8281 cggcccgcag tccgatcatc ggataggcaa tcgccgtgaa gaccagtggc
actgtgagaa
8341 aaagcggcaa ttcggcaatc gttttgccca gaaagtatgt gtcacagcga
taaagtcgac
8401 ttcgggcctc cctcataaaa actggcagct ctgaggtgaa cacctaaatc
gaatcgattc
8461 attagaaagt tagtaaatta ttgaaatgca aatgtattct aaacatgact
tacatttatc
8521 gtggcaaaga cgttttgaaa ggtcatgttg gtcaggaaga ggaagatggc
tccgttgata
8581 ttcatcacac ccacttgcgt gagttgttgg cccaaaaaga tgaggccaat
caagatggca
8641 accatctgca aattaaaatg ttactcgcat ctcattaata ttcgcgagtt
aaatgaaatt
8701 tatttatctt ctgcaaaact ataaactata catctcattg aaaaaaacta
agaagggtgt
8761 ggaatcaggc aattctatct aaaatctagc gaatttgttt ccaagaattg
taagcgttat
8821 atcatttgtt tccactggaa ccactcaccg ttgtctgaat aagtcgcact
tttacgagga
8881 gtggttcctt gagcaccgac agccaggatc gccacaggac cgcccggaac
tgcatgaacc
8941 aggtggcctt gtaggtgtac ccattctccg gctgctccag tggcttctcc
agatttttgg
9001 tggccaacaa ctgctccata tcccgggcta ctttgctaat ggcaaaattg
tcgcatatct
9061 tggcgatccg atcacgggac tcgatctccc gtccgggcac aacggccaac
acctgtacgt
9121 aaaagtccgc cggattgtag ttggtaggac actgggcacc cacgctggat
aggagttgag
9181 atgttatgta atactagata cccttaataa acacatcgaa ctcactagga
aaagaagtcg
9241 acggcttcgc tgggagtgcc caagaaagct accctgccct cggccatcag
aaggatcttg
9301 tcaaagagct caaacagctc ggaagacggc tgatgaatgg tcaggatgac
ggtcttgccc
9361 ttctgcgaca gcttcttcag cacctggacg acgctgtggg cggtaaagga
gtccagtccg
9421 gaggtgggct catcgcagat cagaagcggc ggatcggtta gagcctcgga
ggcgaatgcc

eLife Sciences Publications, Ltd is a limited liability non-profit nonstock corporation incorporated in the State of Delaware, USA with company number 5030732, and is registered in the UK with company number FC030576 and branch number BR015634 at the address Westbrook Centre, Milton Road, Cambridge, CB4 1YG, UK.

9481 agacgcttcc tttctccgcc ggacagacct ttcaccctgc cgggcacacc
gatgatcgtg
9541 tgctgacatt tgctgagcga aagctcctgg atcacctgat ccacgcgggc
cactcgctgc
9601 cgataggtca gatgtcgtgg catccgcacc atggcttgga aaatcaggtg
ttccctggcc
9661 gttagggagc cgataaagag gtcatcctgc tggacatagg cgcacctggc
ctgcatctcc
9721 ttggcgtcca caggttggcc attgagcagt cgcatcccgg atggcgatac
ttggatgccc
9781 tgcggcgatc gaaaggcaag ggcattcagc agggtcgtct ttccggcacc
ggaactgccc
9841 atcacggcca aaagttcgcc cggataggcc acgccgcaaa ctgagtttca
aattggtaat
9901 tggacccttt attaagattt cacacagatc agccgactgc gaatagaaac
tcaccgttct
9961 tgagcaaatg tttcctgggc gccggtatgt gtcgctcgtt gcagaatagt
ccgcgtgtcc
10021 ggttgaccag ctgccgccat ccggagcccg gctgattgac cgccccaaag
atgtccatat
10081 tgtgccaggc ataggtgagg ttctcggcta gttggccgct ccctgaaccg
gagtcctccg
10141 gcggactggg tggccggagc gtgccgtagt tttggcctg cccgaagccc
tggttaatgc
10201 agctctgcga agccgctccg ctgtcaccct gcaatgatag ggatctcaa
atatcaacta
10261 ctagcgttat gctcatctaa ccccgaacaa aaagtacccc gaagtatcct
acgaagtagg
10321 tttatacttt tatttatttt ttgtgcatct aggatcagct taaaatatct
ggttgttata
10381 tttttgtaa aaaagaatat agtcgaaaat gaatgccttt agatgtcttg
atcatgatat
10441 gatctcaaaa attgtcttat atagcgagaa cagctaccag aataatctgt
ttcgtgtcac
10501 tatttgtttg tgcaattgcg gtttgggatt tttgtgggtc gcagttctca
cgccgcatac
10561 aatttgatgt tgcaatcgca gttcctatag atcaagtgaa cttaagatgt
atgcacatgt
10621 actactcaca ttgttcagat gctcggcaga tgggtgtttg ctgcctccgc
gaattaatag
10681 ctcctgatcc tcttggccca ttgccgggat ttttcacact ttcccctgct
tacccaccca
10741 aaaccaatca ccaccccaat cactcaaaaa acaaacaaaa ataagaagcg
agaggagttt
10801 tggcacagca ctttgtgttt aattgatggc gtaaaccgct tggagcttcg
tcacgaaacc
10861 gctgacaaaa tgcaactgaa ggcggacatt gacgctacgt aacgctacaa
acggtggcga
10921 aagagatagc ggacgcagcg gcgaaagaga cggcgatatt tctgtggaca
gagaaggagg
10981 caaacagcgc tgactttgag tggaatgtca ttttgagtga gaggtaatcg
aaagaacctg
11041 gtacatcaaa taccсttgga tcgaagtaaa tttaaaactg atcagataag

eLife Sciences Publications, Ltd is a limited liability non-profit nonstock corporation incorporated in the State of Delaware, USA with company number 5030732, and is registered in the UK with company number FC030576 and branch number BR015634 at the address Westbrook Centre, Milton Road, Cambridge, CB4 1YG, UK.

ttcaatgata
11101 tccagtgcag taaaaaaaaa aaatgttttt tttatctact ttccgcaaaa atgggtttta
11161 ttaacttaca tacatactag gcgcgcccat atgttcggct tgtcgacatg cccgccgtga
11221 ccgtcgagaa cccgctgacg ctgccccgcg tatccgcacc cgccgacgcc gtcgcacgtc
11281 ccgtgctcac cgtgaccacc gcgcccagcg gtttcgaggg cgagggcttc ccggtgcgcc
11341 gcgcgttcgc cgggatcaac taccgccacc tcgacccgtt catcatgatg gaccagatgg
11401 gtgaggtgga gtacgcgccc ggggagccca agggcacgcc ctggcacccg caccgcggct
11461 tcgagaccgt gacctacatc gtcgacggta acatgtgagc aaaaggccag caaaaggcca
11521 ggaaccgtaa aaaggccgcg ttgctggcgt ttttccatag gctccgcccc cctgacgagc
11581 atcacaaaaa tcgacgctca agtcagaggt ggcgaaaccc gacaggacta taaagatacc
11641 aggcgtttcc ccctggaagc tccctcgtgc gctctcctgt tccgaccctg ccgcttaccg
11701 gatacctgtc cgcctttctc ccttcgggaa gcgtggcgct ttctcatagc tcacgctgta
11761 ggtatctcag ttcggtgtag gtcgttcgct ccaagctggg ctgtgtgcac gaaccccccg
11821 ttcagcccga ccgctgcgcc ttatccggta actatcgtct tgagtccaac ccggtaagac
11881 acgacttatc gccactggca gcagccactg gtaacaggat tagcagagcg aggtatgtag
11941 gcggtgctac agagttcttg aagtggtggc ctaactacgg ctacactaga agaacagtat
12001 ttggtatctg cgctctgctg aagccagtta ccttcggaaa aagagttggt agctcttgat
12061 ccggcaaaca aaccaccgct ggtagcggtg gtttttttgt ttgcaagcag cagattacgc
12121 gcagaaaaaa aggatctcaa gaagatcctt tgatcttttc tacggggtct gacgctcagt
12181 ggaacgaaaa ctcacgttaa gggattttgg tcatgagatt atcaaaaagg atcttcacct
12241 agatcctttt aaattaaaaa tgaagtttta aatcaatcta aagtatatat gagtaaactt
12301 ggtctgacag ttaccaatgc ttaatcagtg aggcacctat ctcagcgatc tgtctatttc
12361 gttcatccat agttgcctga ctccccgtcg tgtagataac tacgatacgg gagggcttac
12421 catctggccc cagtgctgca atgataccgc gagacccacg ctcaccggct ccagatttat
12481 cagcaataaa ccagccagcc ggaagggccg agcgcagaag tggtcctgca actttatccg
12541 cctccatcca gtctattaat tgttgccggg aagctagagt aagtagttcg ccagttaata
12601 gtttgcgcaa cgttgttgcc attgctacag gcatcgtggt gtcacgctcg tcgtttggta

eLife Sciences Publications, Ltd is a limited liability non-profit nonstock corporation incorporated in the State of Delaware, USA with company number 5030732, and is registered in the UK with company number FC030576 and branch number BR015634 at the address Westbrook Centre, Milton Road, Cambridge, CB4 1YG, UK.

```
    12661 tggcttcatt cagctccggt tcccaacgat caaggcgagt tacatgatcc
cccatgttgt
    12721 gcaaaaaagc ggttagctcc ttcggtcctc cgatcgttgt cagaagtaag
ttggccgcag
    12781 tgttatcact catggttatg gcagcactgc ataattctct tactgtcatg
ccatccgtaa
    12841 gatgcttttc tgtgactggt gagtactcaa ccaagtcatt ctgagaatag
tgtatgcggc
    12901 gaccgagttg ctcttgcccg gcgtcaatac gggataatac cgcgccacat
agcagaactt
    12961 taaaagtgct catcattgga aaacgttctt cggggcgaaa actctcaagg
atcttaccgc
    13021 tgttgagatc cagttcgatg taacccactc gtgcacccaa ctgatcttca
gcatctttta
    13081 ctttcaccag cgtttctggg tgagcaaaaa caggaaggca aaatgccgca
aaaaagggaa
    13141 taagggcgac acggaaatgt tgaatactca tactcttcct ttttcaatat
tattgaagca
    13201 tttatcaggg ttattgtctc atgagcggat acatatttga atgtatttag
aaaaataaac
    13261 aaataggggt tccgcgcaca tttccccgaa aagtgccacc tgagtc
//
```

eLife Sciences Publications, Ltd is a limited liability non-profit nonstock corporation incorporated in the State of Delaware, USA with company number 5030732, and is registered in the UK with company number FC030576 and branch number BR015634 at the address Westbrook Centre, Milton Road, Cambridge, CB4 1YG, UK.
